# Functional Associations by Response Overlap (FARO), a Functional Genomics Approach Matching Gene Expression Phenotypes

**DOI:** 10.1371/journal.pone.0000676

**Published:** 2007-08-01

**Authors:** Henrik Bjørn Nielsen, John Mundy, Hanni Willenbrock

**Affiliations:** 1 Center for Biological Sequence Analysis, BioCentrum, Technical University of Denmark, Kongens Lyngby, Denmark; 2 Institute of Molecular Biology, Biocenter, University of Copenhagen, Copenhagen, Denmark; Purdue University, United States of America

## Abstract

The systematic comparison of transcriptional responses of organisms is a powerful tool in functional genomics. For example, mutants may be characterized by comparing their transcript profiles to those obtained in other experiments querying the effects on gene expression of many experimental factors including treatments, mutations and pathogen infections. Similarly, drugs may be discovered by the relationship between the transcript profiles effectuated or impacted by a candidate drug and by the target disease. The integration of such data enables systems biology to predict the interplay between experimental factors affecting a biological system. Unfortunately, direct comparisons of gene expression profiles obtained in independent, publicly available microarray experiments are typically compromised by substantial, experiment-specific biases. Here we suggest a novel yet conceptually simple approach for deriving ‘Functional Association(s) by Response Overlap’ (FARO) between microarray gene expression studies. The transcriptional response is defined by the set of differentially expressed genes independent from the magnitude or direction of the change. This approach overcomes the limited comparability between studies that is typical for methods that rely on correlation in gene expression. We apply FARO to a compendium of 242 diverse *Arabidopsis* microarray experimental factors, including phyto-hormones, stresses and pathogens, growth conditions/stages, tissue types and mutants. We also use FARO to confirm and further delineate the functions of *Arabidopsis* MAP kinase 4 in disease and stress responses. Furthermore, we find that a large, well-defined set of genes responds in opposing directions to different stress conditions and predict the effects of different stress combinations. This demonstrates the usefulness of our approach for exploiting public microarray data to derive biologically meaningful associations between experimental factors. Finally, our results indicate that FARO is more powerful in associating mutants in common pathways than existing methods such as co-expression analysis.

## Introduction

Whole-genome expression profiling provides global molecular phenotypes that enable functional analyses of genes and genomes. The amount of public gene expression data is rapidly accumulating due to advances and cost reductions in high-throughput technologies such as DNA microarrays. While reproducibility between identical RNA samples on different microarray platforms between dedicated laboratories is good [1], comparability between studies with independent samples is less satisfactory [2], [3]. Exploitation of the expanding data set has largely been limited to co-expression analysis of genes and comparisons between experimental factors (growth conditions, treatments, specific mutations, etc.) within single studies [4]–[9]. Comparisons between experimental factors have been based on similarities in global expression profiles derived from the signals from all genes on the microarrays. This has enabled clustering of factors to estimate their relatedness. For such analyses, some advanced clustering approaches have been suggested, for example the utility of transcriptional consensus clusters derived from multiple cluster algorithms [8], or incorporation of prior knowledge of gene function [9]. While controllable factors, except the specific factor(s) addressed, typically are kept constant for all experiments within a study, this is rarely true between different studies. Therefore, comparisons of global expression profiles across studies often fail to separate relevant from confounding factors. Fortunately, microarray studies typically include control samples that facilitate the isolation of the effects of factors addressed in the individual studies. Thus, a recent study by Lamb *et al.* [10] presents a method that utilizes fold-change comparisons versus control samples to extract a ‘gene expression signature’ representing an experiment. In this way, experiments were associated based on the significant bias in the ranking of these ‘gene expression signature’ genes.

Sample replicates permit the statistical extraction of differentially expressed genes that are representative of the factor(s) addressed in a study. In this way, the impact of uncontrolled or random differences between samples is reduced. Consequently, we reasoned that relevant associations between experimental factors in different studies can be estimated by first identifying genes responding to a given factor by statistical comparison to control samples within a single study. In contrast to Lamb *et al.* [10], we simply use the overlap in differentially expressed genes in subsequent comparisons between factors of different studies.

Using this approach, we show that response overlaps in genes that are differentially expressed between microarray studies can be used to derive functional associations between experimental factors. We designate this approach ‘Functional Association(s) by Response Overlap’ (FARO). Importantly, FARO is designed to include the possibility that the amplitudes of responses may vary or be reversed, even when closely associated functions are affected. For example, if the proteins encoded by two genes function in a complex, common pathway or network, then overlapping sets of genes may be expected to respond when either gene function is compromised. However, if one protein is a repressor and the other an activator, the resulting responses are likely to affect overlapping gene sets in opposite directions. We further reasoned that while differences in the response direction of the overlapping genes of closely related factors may be expected, consistency in the relative direction, as either congruent or dissimilar, may be descriptive and support their association.

As an example of the approach, we show that FARO between a compendium of 241 *Arabidopsis* gene expression responses from many laboratories and the response of the MAP kinase 4 loss-of-function mutant, *mpk4* [11]–[13], confirms and extends previous studies on the regulatory functions of MAP kinase 4 in pathogen and stress responses [14], [15]. This analysis also demonstrates that FARO enables the prediction of more general biological phenomena including the effects and severities of multiple stresses. In addition, we demonstrate that FARO is superior to co-expression analysis in associating genes according to KEGG [16] and MIPS [17] annotations in the Rosetta Yeast compendium [4]

## Results

### The FARO approach

**Figure 1:**
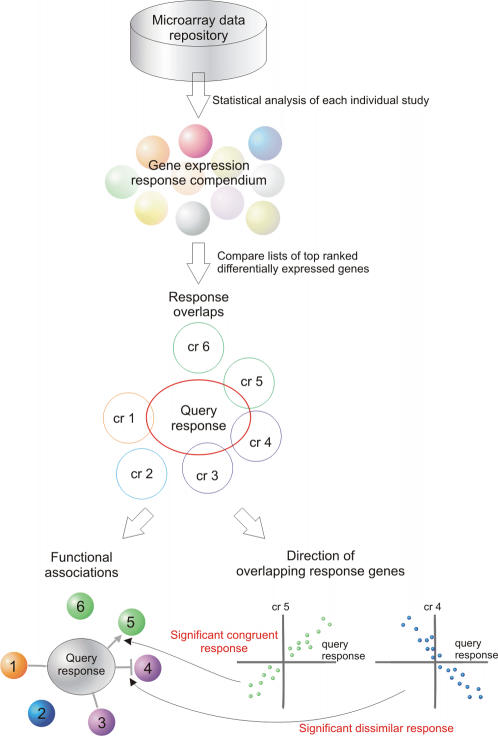
Overview of the FARO method. A large number of gene expression studies from a microarray data repository are analyzed individually, resulting in a compendium of gene expression responses. Each of these responses corresponds to a list of top ranking, differentially expressed genes. A query response, for example a response measured in a new microarray experiment, may then be compared to the compendium responses (cr) and the response overlap in terms of common, differentially expressed genes determined. The strength of an association is determined by the size of the overlap and the result illustrated in a FARO map (bottom right and Figure 2). In the example, the query response demonstrates significant associations to compendium factors 1, 3, 4, and 5. Moreover, it is possible to test if the direction of a response is predominantly dissimilar (factor 4) or congruent (factor 5). This is indicated in the FARO map by a hammerhead or an arrow, respectively.

Transcript profiling experiments are generally designed to assess the effect on gene expression of an experimental factor such as growth condition/stage, treatments, specific mutations, etc. To assign Functional Associations by Response Overlap (FARO) between an experimental factor and the factors assessed in a compendium of gene expression responses, a query response of differentially expressed genes from one study was compared to the responses of the compendium (Figure 1). The associations were ranked by the overlap size and statistical significance was estimated using Fishers exact test [18]. The compendium of gene expression responses was constructed by analyzing the individual studies in a collection of microarray studies to rank genes by their significance of differential expression within each study. The individual experiment was analyzed separately such that individual measurements were only compared directly within a study. Consequently, variations in experimental procedures between experiments have no direct influence on the estimated responses. Assuming that the individual experimental designs were executed carefully, differentially expressed genes represent the response to the factor(s) studied and thus provide an expression phenotype.

**Figure 2:**
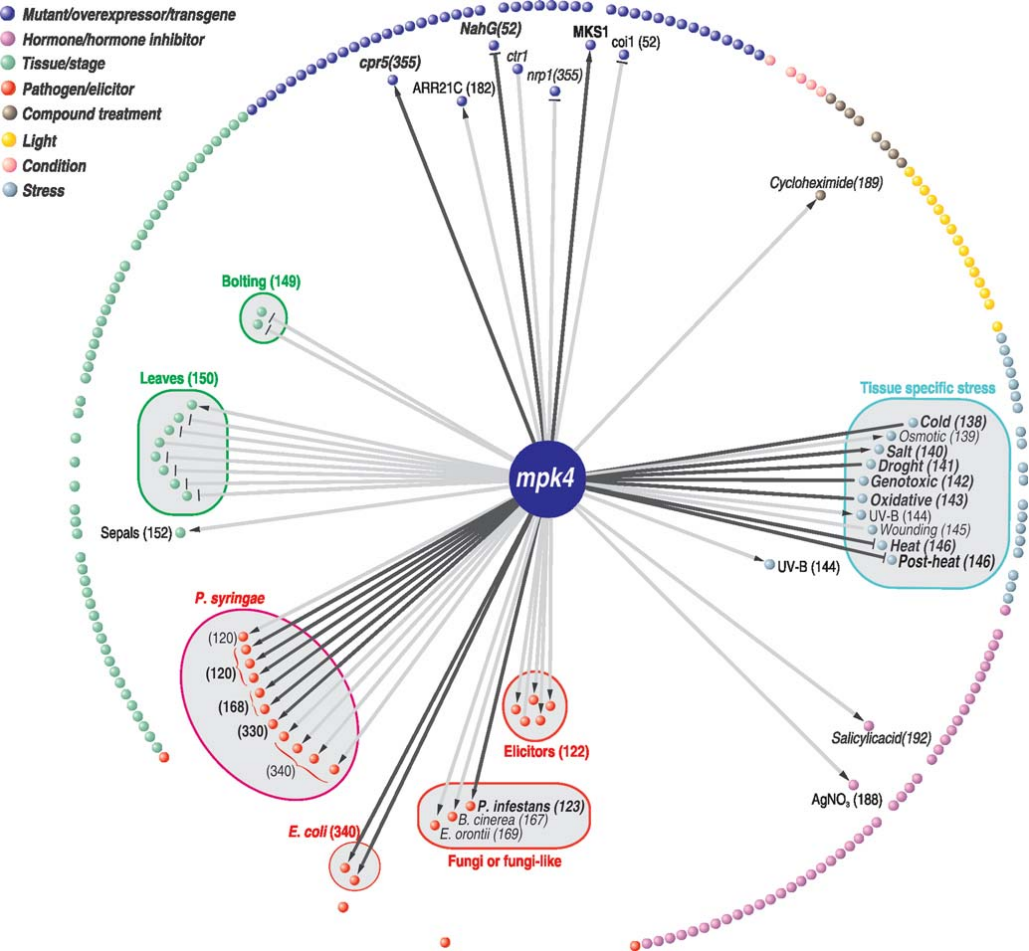
FARO map of the Arabidopsis *mpk4* mutant. The 241 experimental factors (spheres/nodes) in the compendium of responses are divided into 8 categories indicated by different colors. Only edges (lines connecting factors) and names for experimental factors with strong associations to the *mpk4* mutant are shown. Thicker edges and bold factor fonts indicate increasing association strength. Edge arrows or hammerheads, respectively, indicate highly significant congruent or dissimilar (opposite) response direction of the overlapping genes. Significant factors are positioned inside the circle of non-significant factors solely for typographical reasons. NASCArray accession numbers are in parentheses.

### FARO of the *Arabidopsis* MAP kinase 4 mutant

As a test of the approach, we determined FARO of the *Arabidopsis* MAP kinase 4 loss of function mutant (*mpk4*) against a compendium of 241 *Arabidopsis* gene expression profiles representing responses to experimental factors. *mpk4* was chosen because molecular and biochemical studies indicate that MPK4 kinase activity has two opposing functions which require further study [11]–[13]. On the one hand, loss of MPK4 activity leads to the development of systemic acquired resistance to biotrophic pathogens that is dependent upon the phytohormone salicylate. On the other hand, MPK4 activity is required for certain responses to the plant hormones jasmonate and ethylene which induce defences against necrotrophic pathogens and herbivores. A biochemical explanation for these *mpk4* phenotypes is that MPK4 kinase activity, directly or indirectly, is normally required to repress systemic acquired resistance but is also required to induce some responses to jasmonate and ethylene.

The gene expression responses of 241 experimental factors derived from the compendium are represented as colored nodes (spheres) in the FARO map (Figure 2). Edges from the central *mpk4* mutant factor to compendium factors represent the most significant response overlaps. In general, associations above the threshold used (FARO score 60, see Methods) were almost all in agreement with previous molecular and biochemical studies, and only few of the factors below the threshold were previously suggested to be related to the effects of *mpk4* loss-of-function (Supporting Information Table S1).

More specifically, FARO indicated a series of very strong associations between *mpk4* and plants inoculated with virulent and avirulent pathogens. Thus, 16 of the 20 infection studies in the compendium were among the significant associations, while the 4 others were the only 3 insect infestations included and a *Pseudomonas syringae hrpA/fliC* double mutant. The latter is blocked in virulence factor secretion via the type III secretion system due to the *hrpA* mutation, and also cannot produce FliC, a flagellar, pathogen-associated molecular marker [19]. These 4 factors are therefore not expected to associate significantly with *mpk4* or salicylate-dependent systemic acquired resistance. FARO also showed strong associations between *mpk4* and the well-studied mutants *npr1* (non-expressor of pathogenesis-related genes 1, [20], and *cpr5* (constitutive expressor of pathogenesis-related genes 5, [21], both related to systemic acquired disease resistance. These findings are consistent with previous observations that the loss-of-function *mpk4* mutant exhibits constitutive systemic acquired resistance dependent upon salicylate [11]–[13].

MPK4, like other MAP kinases, performs its regulatory function(s) primarily via the phosphorylation of substrate proteins. We have recently shown that the nuclear protein MKS1 is an *in vivo* substrate of MPK4, and that there is significant similarity between the gene expression profiles of the *mpk4* mutant and MKS1 over-expressing plants [12]. In agreement with this, FARO showed that one of the strongest associations to *mpk4 was* to the transgenic MKS1 over-expressor. This may be explained biochemically if the lack of properly phosphorylated MKS1 in *mpk4* mutants, or the excessive accumulation of non-phosphorylated MKS1 in transgenic plants where MPK4 kinase activity is limiting, leads to the development of systemic acquired resistance. Interestingly, FARO also found strong association between *mpk4* and the jasmonate- and coronatine-insensitive 1 (*coi1*) and ethylene constitutive triple response 1 (*ctr1*) mutants, as well as to the ethylene response inhibitor AgNO_3_. The associations between *mpk4* and *coi1*, *ctr1* and AgNO_3_ are in agreement with our findings that MPK4 is required for certain responses to jasmonate and ethylene as well as to salicylate [11], [13]. The significance and possible mechanistic links underlying this may be probed by examining the epistatic relationship between *mpk4* and *ctr1*. This can be determined [22] from the global expression data we recently described both for the *mpk4* and *ctr1* single and for the *mpk4/ctr1* double mutants [13]. This analysis (Supporting Information Text S1) indicated that *mpk4* is, at least in part, epistatic to *ctr1* and again points out the value of comparing differential gene expression responses.

The *Arabidopsis* compendium we used contains 33 studies of responses to 24 phytohormone treatments [23]. Of these, only the response to salicylate associated to *mpk4*, despite the fact that this single study, with only four samples, is among the hormone studies with the least statistical power. While this association is expected due to the elevated levels of salicylate measured in the *mpk4* mutant [11], it illustrates that FARO can overcome limitations in the experimental designs of the underlying studies.

**Figure 3:**
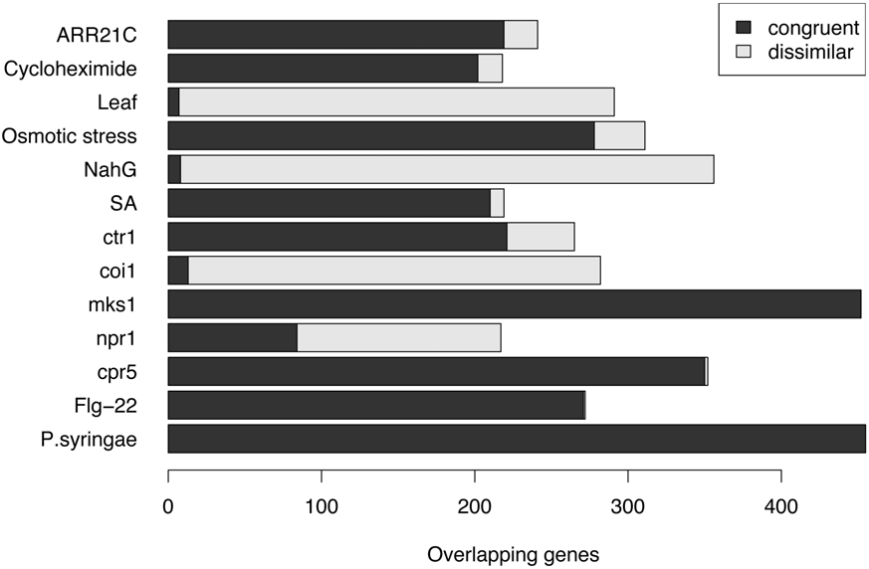
Bar plot of gene expression congruence and dissimilarity. The response overlap between *mpk4* and selected experimental factors are shown. The dark and light areas of the bars indicate congruent and dissimilar gene expression between *mpk4* and an experimental factor, respectively.

The edge arrow- and hammer-heads on the *mpk4* FARO map indicate the predominant congruence or dissimilarity in the direction of the observed responses, some of which are exemplified in Figure 3. For example, the congruence was close to 100% between *mpk4, cpr5*, the MKS1 over-expressor, and pathogen or elicitor-treated plants. In contrast, transgenic plants over-expressing the NahG salicylate hydroxylase, which degrades salicylate to catechol [24], [25], had an inverted response (98% dissimilarity). This very strong association between *mpk4* and *NahG* transgenics confirms the set of genes that are required for *mpk4*- and salicylate-dependent systemic acquired resistance [11]–[13].

In addition to the experimental factors described above, the *Arabidopsis* compendium included 58 organ- or tissue-specific factors. As might be expected, tissues as diverse as pollen, roots or leaves exhibit very large differences in gene expression, and it is therefore an analytical challenge to understand the gene expression profiles which account for their developmental differences and similarities. However, FARO found that, of the 58 tissue-specific factors, the 16 that addressed leaf sections, types or stages all associated to *mpk4* with rank 22 or higher in respect to other tissues (Supporting Information Table S1). This is in keeping with the leaf-specific expression of MPK4 primarily in guard and vascular cells [11]. FARO also found that *mpk4* associated to seedlings at the post-transition and prior-to-bolting stages, both developmental periods in which salicylate levels increase [26]. In addition, the only other tissue with significant associations to *mpk4* was sepals which are photosynthetic and resemble leaves.

### Novel associations to *mpk4*

The FARO described above confirms what we and others have documented about MPK4. However, FARO also identified two other associations to *mpk4*. The first, largely congruent association was to treatment with the protein synthesis inhibitor cycloheximide (CHX). The significance of this association may be consistent with general effects of CHX and the phenotype of *mpk4*. mRNA accumulation in response to CHX often indicates that normal mRNA levels are negatively regulated at the transcriptional and/or post-transcriptional (mRNA stability) levels. Thus, loss of a labile repressor leads to accumulation of its target mRNA(s). Similarly, we previously showed that loss of MPK4 activity leads to derepression of a set of pathogenesis-related genes whose basal expression levels may normally be repressed via plant-specific WRKY transcriptions factors [12]. Thus, it is likely that CHX treatment would induce the accumulation of certain mRNAs that accumulate ectopically in *mpk4*. We note also that while *mpk4* mRNA levels do not change in response to CHX [27], the mRNA of *MKS1*, which encodes an MPK4 substrate [12] whose over-expression is closely associated with *mpk4* by FARO (Figure 2), accumulates strongly (30-fold) as a result of CHX treatment (NASCArray 183). This suggests that steady state levels of MKS1 mRNA are negatively regulated, possibly by feedback from the signaling pathway including MPK4 and MKS1.

The second novel association identified by FARO was between *mpk4* and plants over-expressing the C-terminal, DNA-binding domain of the *Arabidopsis* response regulator 21 (ARR21) driven by the cauliflower mosaic virus 35S promoter (ARR21C [28]; NASCArray 183). ARR21 is a type B ARR with an N-terminal receiver domain thought to regulate the activity of its C-terminal GARP DNA-binding domain. This suggests that ARR21 is or may become nuclear localized, as are both MPK4 and its substrate MKS1 [29]. In contrast to the *arr21* knockout mutant, for which no phenotype was detected [30], over-expression of the constitutively active ARR21C protein results in abnormal development with tissues resembling *in vitro* callus [31]. FARO of ARR21C against the compendium indicated strong associations between ARR21C and zeatin treatments, circadian rhythm, over-expression of the close homolog ARR22 [28], [29], tissue-specific stress responses, as well as inoculation with the oomycete pathogen *Phytophthora infestans.* While this is revealing, a 2^nd^ order FARO, in the form of an analysis for overlap between the *mpk4-arr21* overlap and the compendium, characterized the *mpk4-arr21* association as predominantly related to tissue-specific stress and/or response to *P. infestans* infection.

### Multi-factor FARO

**Figure 4:**
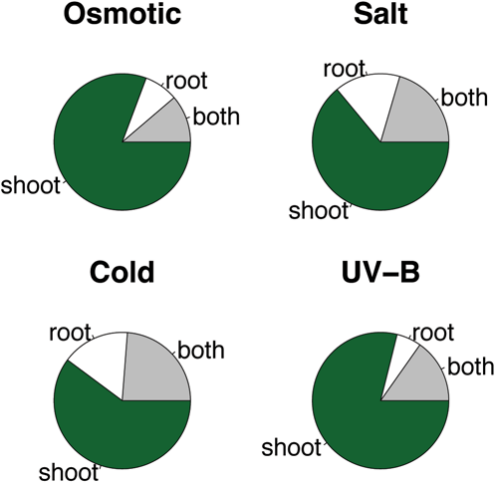
Pie charts showing the fractions of *mpk4* responding genes that are differentially expressed in shoot, root or both in response to osmotic, salt, cold or UV-B stress.

FARO further indicated that MPK4 may be involved in abiotic stress response(s). This was evident from strong associations to a series of stress responses in which organ- or tissue- specificity was a factor (root vs. shoot, NASCArray 137-146). Thus, the overlapping genes demonstrated a strong tendency to respond to stress predominantly in shoots (Figure 4).

**Figure 5:**
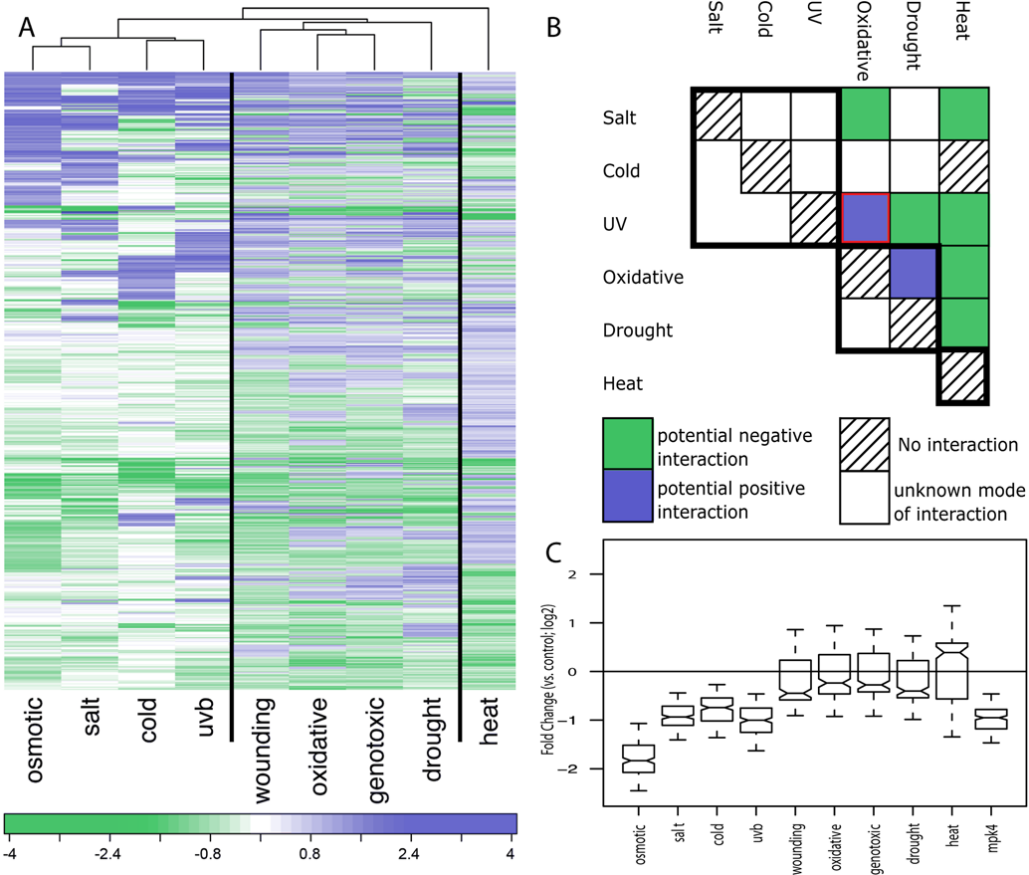
Tissue-specific abiotic stress. A) Expression profiles of the 657 tissue-specific stress response genes for nine different stress conditions. Color bar values correspond to log_2_-fold changes of gene expression values for stress versus controls. The clustering shown is based on congruence between the stress responses. Vertical lines indicate main groups borders. B) Agriculturally important stress combinations (adapted from [33]. Different combinations of abiotic stresses are presented in a matrix to demonstrate potential interactions with agronomic implications. Different interactions are color-coded to indicate potential negative (green, enhanced damage or lethality due to the stress combination) or potential positive (blue, cross-protection due to stress combination) effects. Black borders surround stress types with congruent response (main groups from 5A). Red border surrounds the only inconsistency between the grouping and known interaction (UV vs. Oxidative stress). C) Box plot of the expression profile of the 222 genes that are differentially expressed under all nine tissue-specific stress conditions and in the *mpk4* mutant. Notches indicate 95% confidence interval for the median and whiskers two standard deviations.

This ‘single factor against all’ FARO analysis failed to clearly distinguish between different tissue-specific stress-responses. However, FARO between all 241 factors, creating a 241×241 matrix of associations, revealed a group of tissue-specific stress factors with an extraordinarily large overlap, similar to what has been described as a core environmental stress response in yeast [32]. More specifically, collecting the 1209 most significantly differentially expressed genes (for details, see Methods and Supporting Information Text S2) from each of the nine stress treatments (cold, drought, genotoxic, heat, osmotic, oxidative, salt, UV-B radiation and wounding) resulted in only 1858 different genes. Of these, 657 responded to all nine stress conditions. Interestingly, the response direction of the 657 genes was not conserved between the stress types, which only exhibited an average of 61% congruence (Figure 5A). Interestingly, this observation predicts that plants are unable to provide an adequate response to some combinations of stress. More specifically, clustering of the nine stress conditions, based on congruence of the responding genes, suggests which stress responses are compatible with each other, and which are not. Hence, stress responses that are related may interact positively, while distantly related responses may interact negatively. Figure 5B shows known interactions between agronomically important abiotic stresses. Of these interactions, only the positive interaction between ozone (oxidative stress) and UV radiation may not be explained by the clustering of the stress responses. Such interactions may provide a molecular basis to explain what farmers and breeders have long recognized: combinations of stresses in the field cause the greatest losses to crop productivity worldwide [33].

The extensive overlap between the tissue-specific stress responses further explains why *mpk4* associated to all tissue-specific stress treatments rather than simply to a subset of them. However, the overlap between *mpk4* and all nine stress responses (222 genes), was not a random subset of the stress genes as these 222 genes displayed very similar profiles across the nine stress treatments. To establish this, we randomly sampled 222 genes from the stress response set of genes and calculated the average inter-gene expression profile correlation. This was repeated 10,000 times, and resulted in average correlations ranging from 0.18 to 0.34. In contrast, the subset overlapping with the *mpk4* response had an average correlation of 0.49 (P value ≪ 0.0001). The expression responses of these 222 genes across the nine stress conditions and in the *mpk4* knockout are shown in Figure 5C. These profiles suggest that the *mpk4* knockout may be hyposensitive to osmotic [14], cold, salt [15] and UV-B stress yet either be hypersensitive to heat stress or partly recover from the mutant phenotype under heat stress. The latter will depend on the epistatic relationship between heat response and *mpk4*.

### FARO has cross-platform potential

Exploiting the vast gene expression data in public repositories is often complicated by low cross-platform comparability. To investigate whether the FARO approach could include data generated on different platforms, gene expression responses were extracted from AFGC cDNA studies and compared to our compendium of Arabidopsis gene expression responses based on Affymetrix ATH1 GeneChip data. Genes were linked between the ATH1 GeneChip and the cDNA arrays using locus tags (www.Affymetrix.com), and only genes present on both platforms were compared. Most of these response-overlaps demonstrated good compatibility. More specifically, the cDNA expression profiles of ‘white light treated’ Colombia and Landsberg wild type Arabidopsis plants (NASCArray 250) were highly associated (rank 4 and 3, respectively) with the ‘4 hours white light’ compendium response (NASCArray 124). Moreover, among the top 10 ranking associations to the response compendium, half of the associations were to responses from light treatments, including blue and red light. In addition, the sulfur deficiency cDNA study (NASCArray 271) was highly associated with the corresponding sulfate limitation compendium response (rank 4; NASCArray 171), and the Phytophthora Infestans inoculation study (NASCArray 266) was highly associated with the corresponding compendium response phenotype (rank 6; NASCArray 123). Moreover, cytokinin and gibberellin cDNA studies (NASCArray 288 and 267) were moderately associated (rank 11) with corresponding compendium responses - zeatin and gibberellin (NASCArray 181 and 184). Finally, a cDNA study of ethylene response (NASCArray 227) was highly associated with the compendium response derived from mutants in the EIN2 gene in the ethylene pathway (rank 7 among compendium profile; NASCArray 52). Note that, this last significant overlap was obtained despite the fact that the cDNA array platform only contained ∼2000 genes.

Of nine cDNA experimental factors investigated (IAA induction, NASCArray 197 and NahG vs. WT, NASCArray 312, not shown), the average association rank to a similar compendium experimental factor was 8.2 out of 243 possible factors. In spite of difficulties in linking gene expression information across platforms, quantitative differences in the data from different platforms and the fact that the experiments do not always address identical experimental factors, the above results demonstrate the potential of the FARO approach in bridging between the platforms.

### Benchmarking on the Rosetta Yeast compendium

To validate the performance of FARO in a more quantitative fashion, two benchmarking datasets were created from the Rosetta compendium of yeast gene expression profiles [4]. The Rosetta dataset consists of microarray gene expression data for many yeast deletion mutants and some chemical treatments. Mutants within the Rosetta compendium may be associated by common KEGG category (71 mutant experiments) or by protein-protein interactions annotated in MIPS PPI (30 mutant experiments).

**Figure 6:**
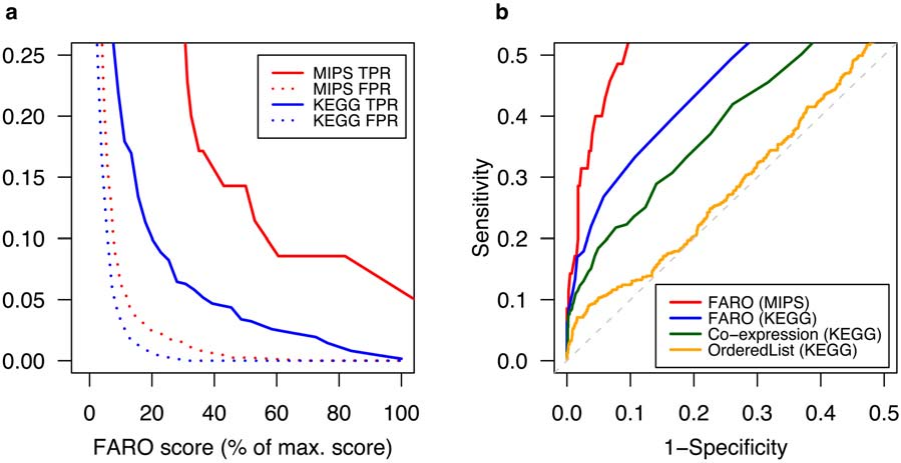
FARO benchmarking. (a) True positive (TPR) and false positive rate (FPR) as a function of the relative FARO score for response overlap (−log10 to the P-value; Fishers exact test). (b) ROC [44] curves of FARO performance.

Within each set, the strength of all associations was estimated by response overlaps. For the KEGG set, 39 correct associations were found that were stronger than any false association. Associations evaluated by use of the manually curated MIPS protein interaction annotations illustrated that the performance on this dataset was even better than for the KEGG dataset (Figure 6a and b). Thus, an extremely high initial true positive to false positive rate was observed in spite of the relatively low number of true associations in the MIPS set (MIPS: 35 true associations out of 436 possible vs. 619 true associations out of 2485 possible in the KEGG dataset). Moreover, the eight chemical treatment experiments included in the Rosetta compendium consistently associated most strongly to mutants in the pathway(s) that the treatments would be expected to affect (Supporting Information Text S3). FARO therefore enriched for true associations. Furthermore, a comparative analysis showed that FARO was superior to a conventional co-expression analysis or a ranking based on the OrderedList Bioconductor package [34] evaluated against corresponding associations in KEGG (Figure 6b).

## Discussion

Functional Association by Response Overlap (FARO) is a robust and conceptually straightforward approach for extracting information on the relatedness of experimental factors (mutants, treatment, experimental condition, etc.) in microarray gene expression experiments made in different laboratories. This enables novel uses of microarray data repositories and offers an advantage over existing analytical methods. We used several methods to appraise the robustness, simplicity and interpretability of FARO.

First, we used FARO to characterize the well-studied plant regulatory mutant *mpk4*. By comparing the result of *mpk4* versus wild type gene expression to a compendium of *Arabidopsis* gene expression responses, we identified associations to a meaningful subset of experimental factors within the compendium. This set of *mpk4* associated factors indicates that the mutant is involved in responses to both virulent and avirulent pathogens, and that the mutant has an expression profile like that of wild type plants treated with the hormone salicylic acid. FARO also indicated that the *mpk4* mutant exhibits a gene expression profile that resembles a shoot-specific stress response. This is in keeping with the finding that mutation or over-expression of putative upstream kinases, which can activate MPK4, are affected in responses to abiotic stresses [15]. The subset of strong *mpk4* associations also contained a series of mutants or treatments affecting responses to the plant hormones ethylene and jasmonic acid that are important for defense regulation. Moreover, a multi-factor FARO analysis indicates that tissue specific responses to various abiotic stress conditions have a very large overlap in terms of differentially expressed genes, but that the response direction varies between the stresses. Clustering the stress conditions based on gene expression congruence predicts the effect and severity of stress combinations, in line with agricultural observations [33]. Hence, FARO can be extended to overview multiple factors. In addition, FARO identified two novel associations between mpk4 and cycloheximide (CHX) treatment and to over-expression of the C-terminal domain of the response regulator ARR21. In short, this characterization of the *mpk4* regulatory mutant was consistent with its previously reported characteristics and with broader knowledge in plant biology. Importantly, the ability of FARO to confirm and extend much of what is known about *mpk4* indicates that FARO will be a powerful tool for elucidating functional associations to more poorly characterized mutants.

Second, we extended this analysis to include the comparison of a series of cDNA microarray studies to our Affymetrix ATH1 GeneChip based Arabidopsis Compendium. This indicated that FARO is also applicable for cross-platform analyses, even including smaller arrayed gene sets.

Third, we used the Rosetta Yeast compendium [4] to produce a more quantitative benchmarking of FARO. These analyses demonstrated that FARO had a remarkable ability to re-extract the groupings and protein interactions specified in both the KEGG and MIPS annotations. In this respect, FARO was clearly superior to the commonly applied method of co-expression analysis for identifying genes co-regulated in response to different experimental factors. Moreover, as an alternative to using the overlap size, several statistical approaches have been proposed for comparing lists of genes from microarray experiments [34], [35]. These methods use the rank of the genes in the respective lists to identify a common gene set and estimate the significance of this by permutations. However, we show that the much simpler FARO method performed significantly better than the OrderedList method (Lottaz et al., 2006) in identifying functional associations (Figure 6).

For all of the analyses described, FARO demonstrated very high robustness toward experimental noise. Much of this robustness is due to the indirect comparison of individual experimental results. That is, the FARO approach restricts direct comparisons between microarrays to within single experiments or studies, and only the outcomes of the statistical analyses in the form of differentially expressed genes are compared between experiments. Hence, FARO benefits from the care taken by experimentalists to ensure comparability within their individual experimental designs. In addition, the extraction of differentially expressed genes serves as a feature selection step, enriching for genes that are characteristic for the given experimental factor. This reduces the amount of noise in comparisons between factors and consequently contributes significant robustness of the analysis.

Weakly designed or poorly conducted experiments may result in poorly defined lists of responding genes and tend to result in a smaller overlap than otherwise expected for truly associated factors. Thus, a poor quality experiment may result in false negatives, but is unlikely to result in false positive associations. Only experiments with undescribed and/or uncontrolled confounding experimental factors may result in highly significant, misleading associations. Similarly, the FARO approach may not be able to show strong associations to an experimental factor that only results in expression changes of a few genes. The probable cut-off in terms of top ranking genes used may need to be adjusted for such factors.

While clustering schemes based on whole-genome profile comparisons may also provide functional predictions for individual genes [8], [36], none of these schemes are as easily interpretable as FARO. Although the interpretation of a FARO requires an understanding of the biological system analyzed, FARO offers an advantage over more abstract methods since FARO results may be further dissected into the individual genes that constitute the overlap. Thus, interpretations of FARO results can be investigated by any systematic analysis that may be applied to the list of overlapping response genes. Examples are GO-term over-representation, chromosomal location bias, or even 2^nd^ order FARO analyses. Consequently, the annotation of the overlapping genes may directly facilitate an interpretation of the functional association. Moreover, the congruence or dissimilarity in response directions of the overlapping genes may clarify relationships indicated by the association.

The results obtained here for two model organisms, *Arabidopsis* and yeast, indicate the usefulness of our method for exploiting available microarray data for deriving functional associations. Given the amount of public microarray data, the applications for this method may be extended to the characterization of other species, including pathogens and humans. For example, the same approach might be useful for associating cancer gene expression response phenotypes to a compendium of cancer responses and cancer treatment responses for diagnostic purposes. Consequently, this study, together with that of Lamb et al. [10], points out the multitude of issues that can be addressed by associations between transcriptional responses. Furthermore, we have benchmarked the inherent sensitivity and robustness of deriving associations from such responses. We further note that while FARO is conceptually simpler than the method of Lamb *et al.* [10], FARO is able to associate factors not related by a congruent or dissimilar response, but only by the mere overlap in responding genes. The important relations found between abiotic stress responses in Arabidopsis exemplify this.

Apart from being more powerful, an advantage of FARO over approaches utilizing co-expression measurements is the ability of FARO to associate not only genes or proteins, but any type of factors that may be experimentally addressed, including drug treatments and disease stages. Moreover, associations between analyzed experimental factors may be used to reveal clusters of factors in a functional association network that may be integrated with other data sources. Consequently, FARO enables exogenous factors to be associated directly to genotypes and as such unites bottom-up and top-down analytical approaches in a single association scheme.

## Methods

### *Arabidopsis* Compendium of Gene Expression Responses

The Nottingham *Arabidopsis* Stock Center (NASC) compendium of global expression data (http://affymetrix.arabidopsis.info/) is a repository of microarray gene expression data from numerous studies [37]. From this repository, we selected the AffyWatch II and III collection, including data from the AtGenExpress consortium and 29 focused studies from various laboratories as well as three of our own studies: the MAP kinase 4 (*mpk4*) knockout mutant [13], the MAP kinase 4 substrate 1 (MKS1) over-expressor [12], and the ethylene constitutive triple response 1 mutant (*ctr1*) [13], [38]. From the Arabidopsis Functional Genomics Consortium (AFGC) microarray project data collection, we also selected six cDNA studies for cross-platform compatibility benchmarking. A comprehensive list of the studies and their experimental factors is provided in Supporting Information Table S1. The compendium can be downloaded from: www.cbs.dtu.dk/databases/PlantExpr/

Experimental factors were manually extracted from the description files, and each study was analyzed separately with regard to the experimental factors in its design. Microarray data was pre-processed by RMA [39], [40]. Appropriate statistical tests (T-test, ANOVA or Fourier analysis) were used to extract lists of genes ranked by their significance of differential expression for the 241 compendium factors as well as for the *mpk4* factor. In total, more than 1700 microarray experiments were analysed.

### KEGG and MIPS

Two benchmarking sets were created by extracting mutants experiments that can be associated to other mutant experiments, within the Rosetta Yeast Expression Profile Compendium [4], by common annotation in the Kyoto Encyclopedia of Genes and Genomes (KEGG: http://www.genome.jp/kegg/), or by protein-protein interactions annotated in MIPS PPI (from the manually curated, comprehensive *Saccharomyces cerevisiae* protein-protein interaction database http://mips.gsf.de/). These sets respectively comprised 71 and 30 mutant experiments. The KEGG category cell cycle was assigned to six additional genes recently found to be involved in yeast cell cycle [41]. For the KEGG dataset, 619 proteins were associated by common KEGG category, among 2485 possible associations between mutants. For the MIPS dataset, 35 associations by MIPS interactions were present among 435 possible associations between mutants.

### Statistical Significance

The statistical significance of the response overlap, in terms of overlap in differentially expressed genes, was estimated using Fisher's exact test [18]. The statistical significance of congruence in the up or down regulation of overlapping genes was determined using an exact test in the binomial distribution [42], [43], where the hypothesized probability of success was fixed at 0.5.

With regard to the optimal number of top ranking genes to include in a comparison between experimental factors, we found it optimal to include genes that ranked higher than the median number of significant genes in the compendium studies at a significance level lower than 0.05. While the inclusion of an increasing number of response-specific genes will strengthen a true response overlap signature, including too many genes may disturb the expression associations. Thus, the 1209 most significantly differentially expressed genes were used for the *Arabidopsis* compendium, and the 57 and 170 most significantly differentially expressed genes were used for the KEGG and MIPS benchmarking datasets, respectively. Additional considerations regarding the number of genes to include in a FARO are discussed in Supporting Information Text S2.

## Supporting Information

Text S1 mpk4 is epistatic to ctr1.
(0.07 MB DOC)

Text S2 Significant genes in a FARO analysis.
(0.69 MB DOC)

Text S3 Treatment experiments in the Rosetta Yeast compendium.
(0.02 MB DOC)

Table S1 (0.13 MB XLS)
