# Supplementary material for: Functional Associations by Response Overlap (FARO), a Functional Genomics Approach Matching Gene Expression Phenotypes
Source: PLoS One. 2007 Aug 1;2(8):e676. doi: 10.1371/journal.pone.0000676 (PMC1924877; doi:10.1371/journal.pone.0000676)
Supplement: Text S1 — mpk4 is epistatic to ctr1. (0.07 MB DOC) [file pone.0000676.s001.doc]

## *mpk4* is epistatic to *ctr1*

Epistasis is used here to describe the relationship between two non-allelic mutants that have different phenotypes. If the phenotype of the double mutant is similar to that of one of the single mutants, then that mutant is epistatic to the other. If gene expression differences between a mutant and wild type are considered phenotypic traits, the phenotypic differences between the single *mpk4* and *ctr1*, and the double *mpk4/ctr1* mutants can be measured by the number of shared genes in a top list (*n*=1209) of differentially expressed genes between the mutants. Given the size of the *Arabidopsis* transcriptome, approximately, 64 shared genes are expected at random. However,

265 genes are shared by *mpk4* and *ctr1*,

433 genes are shared by *ctr1* and *mpk4/ctr1*,

716 genes are shared by *mpk4* and *mpk4/ctr1*.

By this measure, *mpk4* is apparently much closer than *ctr1* to *mpk4/ctr1* and this indicates that *mpk4* is, at least in part, epistatic to *ctr1.* Alternatively, this observation could be explained as an effect of the mix of the two single mutations in which the relatively smaller distance between *mpk4* and *mp4/ctr1* is due to a higher number of genes being affected by the *mpk4* than by the *ctr1* mutation*.* This hypothesis can be tested by examining the expression levels of genes differentially expressed in both *ctr1* and *mpk4* compared to *mpk4/ctr1*. More specifically, an epistasis analysis normally requires that the mutant phenotypes are antagonistic. In the case of *mpk4* and *ctr1*, however, most (83%) of the genes that are significantly differentially expressed in both mutants are differential expressed in the same direction (congruent) relative to wt. Despite this fact, the analysis is possible because their gene expression phenotypes can be measured quantitatively. The density plot below (Fig. S1) shows the expression level differences between the *mpk4/ctr1* double mutant and both *mpk4* (black) and *ctr1* (red). Furthermore, the expression of 209 of these genes (*n*=265) in *mpk4/ctr1* is more similar to that in *mpk4* than in *ctr1*. Clearly, *ctr1* is more different from the double mutant than *mpk4* is. Therefore, *mpk4* is epistatic to *ctr1*, at least in respect to a subset of *ctr1* response genes*.*


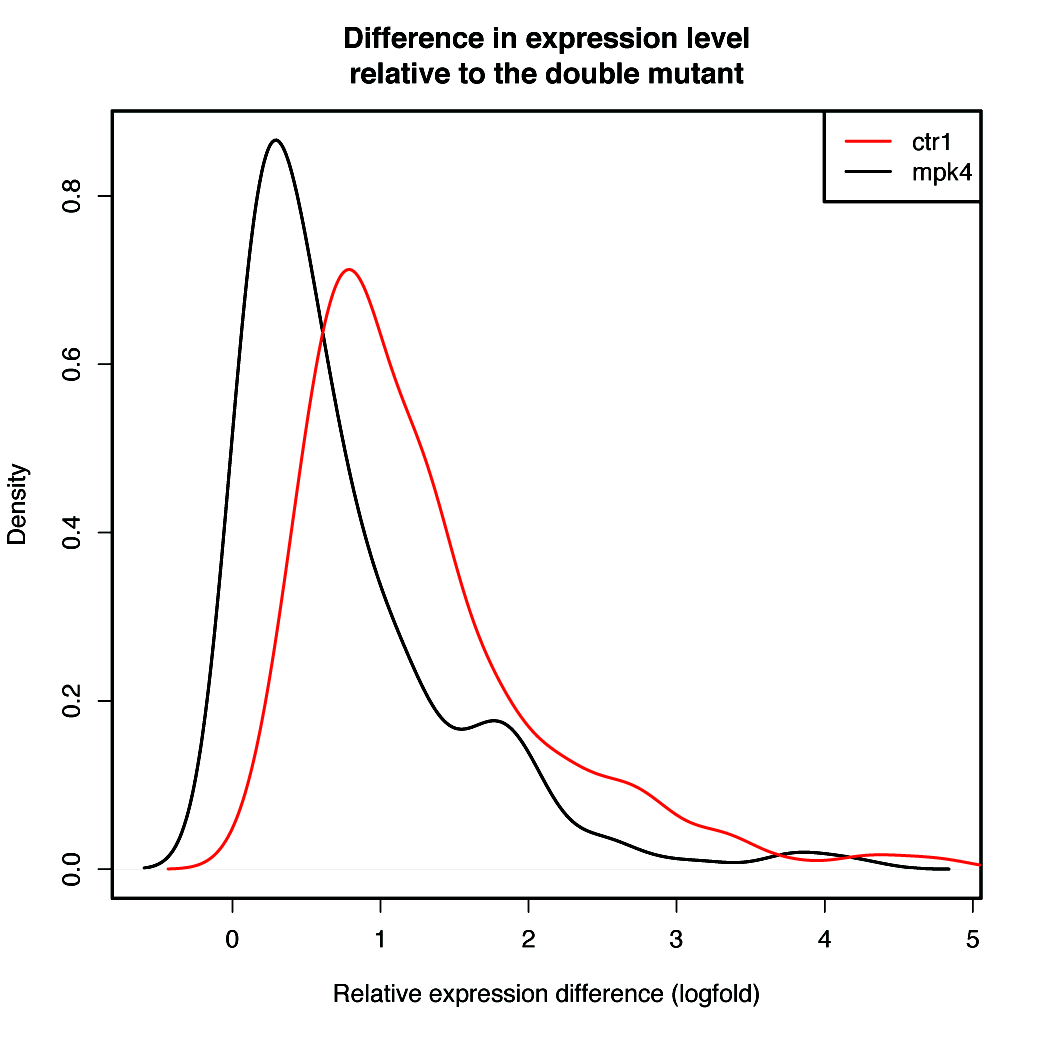


Density plot of the distribution of expression differences between single and double mutants for the 265 genes that are differentially expressed in both *mpk4* and *ctr1* (higher values signify a bigger difference between a mutant and the double mutant).
