# Supplementary material for: Functional Associations by Response Overlap (FARO), a Functional Genomics Approach Matching Gene Expression Phenotypes
Source: PLoS One. 2007 Aug 1;2(8):e676. doi: 10.1371/journal.pone.0000676 (PMC1924877; doi:10.1371/journal.pone.0000676)
Supplement: Text S2 — Significant genes in a FARO analysis. (0.69 MB DOC) [file pone.0000676.s002.doc]

**Significant genes in a FARO analysis**

Limiting the number of false positives is often a major consideration in determining the number of significantly differentially expressed genes included in a microarray study. However, in a FARO analysis, the exclusion of false positives is less important than the inclusion of true positives. Therefore, the cutoff for the microarray studies that serve as input to a FARO analysis can be less strict than the cutoff used in a traditional microarray analysis. In the analysis of the Rosetta compendium of yeast gene expression profiles [1], we found it optimal to include genes that ranked higher than the median number of significant genes (*p* < 0.05, uncorrected) in respect to associating factors according to KEGG and MIPS. Hence, in the KEGG and MIPS benchmarking datasets, the 57 and 170 most significantly differentially expressed genes were used, respectively. When applying this approach (median number of genes that are significant at alpha=0.05) to the *Arabidopsis* compendium, 1209 most significant genes should be included. However, further analyses demonstrated that the exact number of genes included was not very critical to the outcome of the FARO, as its performance was robust over a large range (Fig. S2). Thus, a comparison of FARO analyses including a variable number of top significantly differentially expressed genes demonstrated that the rank of an *mpk4* association differed very little when using lists in the interval between 900 to 4000 genes. This was especially true for factors that associated strongly to *mpk4*. It should be noted that the ‘optimal’ cutoff may depend on the query factor and it is recommended that the cutoff be adjusted depending on the number of genes impacted by this factor.


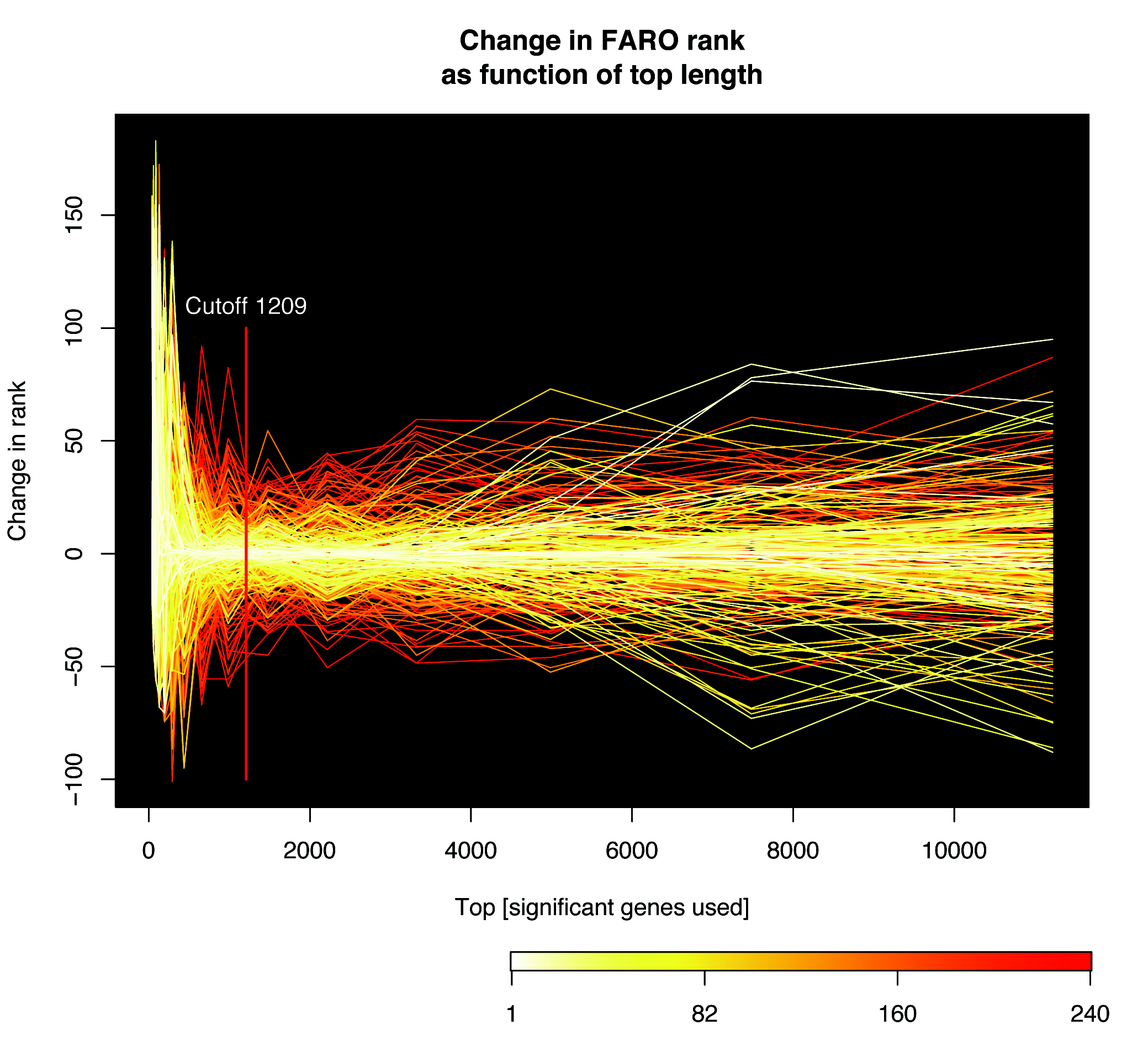


**Welder plot**. Change in FARO rank as a function of the number of significant genes included. Lines indicate the change in rank of the 241 FARO factors in the *Arabidopsis* compendium between a series of most significant gene lists. Note that the length of the list increases exponentially to ensure comparability between the rank differences. The factors are colored according to their rank at ‘Top 1209’ genes. For example, factors ranking in the top (most significant) for 1209 genes are illustrated in white and vary only little in rank except in the lower range, that is, when including only very few top ranking genes in the FARO analysis.

**References**

1. Hughes, T.R. et al. Functional discovery via a compendium of expression profiles*. Ce*l**l 1**02, 109-26 (2000).
