# Supplementary material for: Functional Associations by Response Overlap (FARO), a Functional Genomics Approach Matching Gene Expression Phenotypes
Source: PLoS One. 2007 Aug 1;2(8):e676. doi: 10.1371/journal.pone.0000676 (PMC1924877; doi:10.1371/journal.pone.0000676)
Supplement: Text S3 — Treatment experiments in the Rosetta Yeast compendium. (0.02 MB DOC) [file pone.0000676.s003.doc]

## Treatment experiments in the Rosetta Yeast compendium

The eight treatment experiments from the Rosetta compendium of yeast gene expression profiles [1] with known target gene or biological pathway, were used to investigate if treatment targets could be identified by the FARO approach. For this, the KEGG benchmarking dataset was considered together with additional experiments of mutants in the known target genes.

Convincing results were obtained for all eight treatments. For example, the three top ranking associations (hmg1, HMG2 and ERG11) of the ergosterol biosynthesis inhibitor lovastatin [2] were all members of the ergosterol pathway. For itraconazole, a specific inhibitor of ERD11, the three most significant response overlaps were with erg3, hmg1 and ERG11 [3], all in the sterol biosynthesis pathway. For yeast treated with the translation elongation inhibitor cycloheximide, the most significant response overlap was with yef3 (Translation elongation factor eEF3), and for samples treated with the DNA synthesis inhibitor hydroxyurea (HU), the most significant response overlap was with rnr1 which is important in DNA replication. Tunicamycin, glucosamine and 2-deoxy-D-glucose impact yeast cell wall function [1] and among the highest ranking response overlaps (top 2 for tunicamycin and glucosamine, top 4 for 2-deoxy-D-glucose) were *gas1* and *fks1*, both of which are cell wall-related mutants [1]. Moreover, these 3 compounds had significant response overlaps with each other, indicating that they all have very similar effects in yeast.

**References**

1. Hughes, T.R. et al. Functional discovery via a compendium of expression profiles. *Cell* **102**, 109-26 (2000).

2. Alberts, A.W. et al. Mevinolin: a highly potent competitive inhibitor of hydroxymethylglutaryl-coenzyme A reductase and a cholesterol-lowering agent. *Proc Natl Acad Sci U S A* **77**, 3957-61 (1980).

3. Lamb, D.C. et al. Purification, reconstitution, and inhibition of cytochrome P-450 sterol delta22-desaturase from the pathogenic fungus Candida glabrata. *Antimicrob Agents Chemother* **43**, 1725-8 (1999).
